# Supplementary material for: Is concern about young people's anti-social behaviour associated with poor health? cross-sectional evidence from residents of deprived urban neighbourhoods
Source: BMC Public Health. 2012 Mar 20;12:217. doi: 10.1186/1471-2458-12-217 (PMC3364159; doi:10.1186/1471-2458-12-217)
Supplement: Additional file 1 — Variables used in PASB analysis. [file 1471-2458-12-217-S1.DOC]

**Additional File 1 – Variables used in PASB analysis**

**Dependent variables**

***Teenager problem*** variable appears as item ‘j’ on the following list of questions about neighbourhood problems taken from the 2006 GoWell survey.

*Question:* **For each of the following statements, could you tell me whether you think that each of these is a serious problem, a slight problem or not a problem in your local neighbourhood?** [SINGLE CODE ONLY FOR EACH. ROTATE ORDER OF ASKING STATEMENTS].

|  | | Not a problem | Slight problem | Serious problem | Don’t know |
| --- | --- | --- | --- | --- | --- |
| a | Vandalism, graffiti and other deliberate damage to property or vehicles | 1 | 2 | 3 | 4 |
| b | Violence including assaults and muggings | 1 | 2 | 3 | 4 |
| c | People being insulted, pestered or intimidated in the street | 1 | 2 | 3 | 4 |
| d | Noisy neighbours or loud parties | 1 | 2 | 3 | 4 |
| e | Abandoned or burnt out cars | 1 | 2 | 3 | 4 |
| f | People being attacked or harassed because of their skin colour, ethnic origin | 1 | 2 | 3 | 4 |
| g | People using or dealing drugs | 1 | 2 | 3 | 4 |
| h | People being drunk or rowdy in public places | 1 | 2 | 3 | 4 |
| i | Gang activity | 1 | 2 | 3 | 4 |
| j | Teenagers hanging around on the street | 1 | 2 | 3 | 4 |
| k | Nuisance neighbours or problem families | 1 | 2 | 3 | 4 |
| l | Dogs roaming about / dog fouling / barking | 1 | 2 | 3 | 4 |
| m | Rubbish or litter lying around | 1 | 2 | 3 | 4 |
| n | Vacant or derelict buildings and sites | 1 | 2 | 3 | 4 |
| o | Tensions between Protestants and Catholics | 1 | 2 | 3 | 4 |
| p | House break-ins / burglary | 1 | 2 | 3 | 4 |
| q | Untidy gardens | 1 | 2 | 3 | 4 |

Note: in each case , the ‘not a problem’, ‘slight problem’ and ‘don’t know’ responses were collapsed to create a binary variable comparing ‘not a serious problem’ with ‘serious problem.’ All 6008 participants answered these questions.

***SF-12 version 2 physical and mental health composite scores*** are computed using the scores of 12 questions from a validated questionnaire and range from 0 to 100, where a zero score indicates the lowest level of health measured by the scales and 100 indicates the highest level of health. 5991 of the 6008 participants answered all 12 questions. The remaining 17 participants were treated as missing data.

**Independent variables**

| **No.** | **ORIGINAL QUESTION** | **VARIABLES USED IN ANALYSIS** |
| --- | --- | --- |
| 1 | **In the past 12 months, how many times have you seen or spoken to a doctor from your practice regarding your own health or wellbeing?**   1. None 2. Once or twice 3. Three or four times 4. Five or six times 5. Seven times or more 6. Refused | **Number of GP visits during (last 12 months).**  None = a  1 to 6 visits = b, c, d  7 or more = e  Refused counted as missing (n=3) |
| 2 | **And in the past 12 months, have you spoken to a GP or family doctor on your own behalf, either in person or by telephone about being anxious or depressed or about a mental, nervous or emotional problem (including stress)?**   1. Yes 2. No 3. Refused | **Number of GP visits for a psychological issue (last 12 months)**  None = b  1 or more = a  Refused counted as missing (n=64) |
| 3 | **Are you**   1. Male? 2. Female? | **Sex**  Male = a  Female = b  No missing data. |
| 4 | **To which of these groups do you consider you belong?**   1. White Scottish 2. White British 3. White English 4. White Northern Irish 5. White Welsh 6. White Irish 7. White Any other White background (please write in) 8. Mixed - White and Black Caribbean 9. Mixed - White and Black African 10. Mixed - White and Asian 11. Mixed - Any other Mixed background (please write in) 12. Asian or Asian British - Indian 13. Asian or Asian British - Pakistani 14. Asian or Asian British - Bangladeshi 15. Asian or Asian British - Any other Asian background 16. Black or Black British - Caribbean 17. Black or Black British - African 18. Any other Black background (please write in) 19. Chinese 20. Other (please write in) 21. Do not wish to disclose | **Ethnic group**  White British = a to e  Other = f to t  Refused counted as missing (n=9) |
| 5 | **What is the relationship of each household member to you?** HOUSEHOLD GRID IN WHICH INFORMATION ABOUT EACH HOUSEHOLD MEMBER WAS RECORDED.   1. Spouse/partner/cohabite 2. Son/Daughter (including step/adopted) 3. Grandson/granddaughter (including step/adopted) 4. Parent/parent in-law 5. Other relative 6. Other non-relative | **Household structure**  Cohabiting = a  Single = b to f  Refused counted as missing (n=53) |
| 6 | **Which of the following age bands apply?**   1. LESS THAN 16 YRS 2. 16 – 17 3. 18 – 24 4. 25 – 39 5. 40 – 54 6. 55 – 64 7. 65 PLUS 8. DON’T KNOW 9. REFUSED   This question was asked for the participant. It was also asked for all the other household members to find out if there were children in the house. | **Age group**  65 or older = g  55 to 64 = f  40 to 54 = e  25 to 39 = d  16 to 24 = a to c  Don’t know and refused counted as missing (n=65)  **Living with children**  No = b to g  Yes = a  Don’t know and refused counted as missing (n=36) |
| 7 | **Can you tell me what is the highest level of educational qualifications you’ve obtained?** | **Educational qualifications**  1 or more = c to k  None = a, b, l  Refused counted as missing (n=20) |
|  | 1. School leaving certificate |
|  | 1. O Grade, Standard Grade, GCSE, CSE, or equivalent – grades D to F |
|  | 1. O Grade, Standard Grade, GCSE, CSE, or equivalent – grades A to C |
|  | 1. Higher Grade/A Level, AS Level, Advanced Senior cert, CSYS or equivalent |
|  | 1. GSVQ or SVQ Level 1 or 2, BTEC First Diploma, City and Guilds Craft or equivalent |
|  | 1. GSVQ or SVQ Level 3, ONC, OND or SCOTVEC National Diploma, City and Guilds Advanced Craft, RSA Advanced Diploma or equivalent |
|  | 1. Apprenticeships or trade qualification |
|  | 1. HNC, HND, SVQ levels 4 or 5, RSA Higher Diploma or equivalent |
|  | 1. First degree, Higher Degree |
|  | 1. Other technical or business qualification / certificate |
|  | 1. OTHER |
|  | 1. None of these |
| 8 | **Looking at the card, which option best describes how often you find it difficult to meet the cost of gas, electricity and other fuel bills?**   1. Very often 2. Quite often 3. Occasionally 4. Never 5. Don’t know 6. Not applicable | **Problems paying bills**  Never = d to f  Sometimes = a to c  No missing data. |
| 9 | **How much do you agree or disagree with the following statements? *In each case the response options are as follows…***   1. Strongly agree 2. Agree 3. Neither agree nor disagree 4. Disagree 5. Strongly disagree 6. Don’t know | **Home Psychosocial Environment:**  **feel safe at home; feel in control at home; feel privacy at home; self-esteem from home.**  Yes = a to c  No = d to f  No missing data |
| 9.1 | **I feel I have privacy in my home** |
| 9.2 | **I feel in control of my home** |
| 9.3 | **My home makes me feel that I’m doing well in my life** |
| 9.4 | **I feel safe in my home** |
| 10 | **How safe would you feel walking alone in this neighbourhood after dark?**  SINGLE CODE ONLY. IF RESPONDENT STATES THAT NEVER WALKS ALONE AFTER DARK THEN PROMPT:  **Is that because you don’t feel safe?**  IF YES PROBE:  **Would you say you feel a bit unsafe or very unsafe?** CODE ACCORDINGLY   1. Very safe 2. Fairly safe 3. Neither safe nor unsafe 4. A bit unsafe 5. Very unsafe 6. DO NOT PROMPT: Never walk alone after dark | **Safe neighbourhood**  Yes = a to c  No = d to f  No missing data |
| 11 | **To what extent do you agree or disagree with the following statements… *In each case the response options are as follows…***   1. Strongly agree 2. Agree 3. Neither agree nor disagree 4. Disagree 5. Strongly disagree 6. Don’t know | **Neighbourhood Psychosocial Environment: informal controls in neighbourhood; good neighbourhood reputation; tolerant neighbourhood; trust neighbours; efficacy; self-esteem from neighbourhood.**  Yes = a to c  No = d to f  No missing data |
| 11.1 | **It is likely that someone would intervene if a group of youths were harassing someone in the local area** |
| 11.2 | **Many people in Glasgow think this neighbourhood has a bad reputation** |
| 11.3 | **This neighbourhood is a place where people from different backgrounds get on well together?** |
| 11.4 | **Someone who lost a purse or wallet around here would be likely to have it returned without anything missing** |
| 11.5 | **On your own, or with others, you can influence decisions affecting your local area** |
| 11.6 | **Living in this neighbourhood helps make me feel that I’m doing well in my life** |
| 12 | **To what extent do you feel that you belong to this neighbourhood?**   1. Very strongly 2. Fairly strongly 3. Not very strongly 4. Not at all | **Neighbourhood belonging**  Yes = a and b  No = c and d  No missing data |
| 13 | **On the whole, do you think that over the past two years, this area has got better or worse to live in or has it stayed the same?**   1. The area has got better 2. The area has stayed the same 3. The area has got worse 4. Have lived here less than two years 5. Don’t know | **Neighbourhood decline in last 2 years**  Not decline = a, b, e  Decline = c  Not lived here 2 years = d  No missing data |
| 14 | **How long in total have you lived in this area?**   1. Under 1 year 2. 1-2 years 3. 3-5 years 4. 6-10 years 5. 11-20 years 6. 21+ years 7. Don’t know/cant recall 8. Refused | **Length of residence in neighbourhood (years)**  <2 = a and b  Over two years = c to f  Don’t know/refuse count as missing data (n=173) |
| 15 | **In a typical week, on how many days do you do go for a walk around the neighbourhood**   1. **0** 2. **1** 3. **2** 4. **3** 5. **4** 6. **5** 7. **6** 8. **7** | **Walk around neighbourhood**  <weekly = a  At least weekly = b to h  Refused counted as missing (n=51) |
| 16 | **Thinking about how often you personally contact your relatives, friends and neighbours but not counting the people you live with – how often do you do any of the following? *In each case the response options are as follows…***   1. Most days 2. Once a week or more 3. Once or twice a month 4. Less often than once a month 5. Never 6. Don’t know | **Social contact and support: speak to neighbours; meet with relatives; meet with friends.**  At least weekly = a, b  <weekly = c to e  No missing data |
| 16.1 | **Speak to neighbours** |
| 16.2 | **Meet up with relatives** |
| 16.3 | **Meet up with friends** |
| 17 | **Thinking now about your relatives, friends and neighbours outside your home, can you tell me around how many people could you ask to give you advice and support in a crisis**   1. None 2. One or two 3. More than two 4. Would not ask 5. Don’t know | **Social support**  Somebody = b, c  Nobody = a, d, e  No missing data |

# Note: Questions 1, 3, 5 and 9 derived from SHARP (Scotland's Housing And Regeneration Project) study [1]. Question 2 derived from Health Survey for England [2]. Questions 10, 11.1, 11.4, 11.5, 12 and 13 derived from Home Office Citizenship Survey [3]. Question 7 derived from National Evaluation of the New Deal for Communities Household Survey Questionnaire [4]. Question 11.3 derived from The West of Scotland Twenty-07 Study[5-6]. Questions 16 and 17 derived from Office of National Statistics Social Capital Module [7, 8]. Questions 4, 8 and 14 derived from Glasgow Housing Association social survey conducted by BMG research. Questions 6, 11.2, 11.6 and 15 were developed by the GoWell team. Also note that we have identified the sources of questions we used to populate the GoWell Questionnaire but it is possible that some questions may have appeared or originated in other surveys besides those listed in the above table. Survey questionnaires were located online (e.g. using Survey Question Bank: <http://surveynet.ac.uk/sqb/introduction.asp>), or through direct contact with researchers.

Comparisons of key demographic variables found that ‘missing’ participants had similar gender, education age and marital status to participants included in the analysis. The ‘missing’ participants had a slightly higher mean SF12v2 physical health score (1.5 points greater than the mean score for included participants) and likelihood of being a parent, reporting problems paying bills and of not identifying their ethnicity as white UK. Both groups had similar outcomes for mean SF12v2 mental health scores and for reporting that teenagers were a serious problem.

References

1. Petticrew M, Kearns A, Mason P, Hoy C: **The SHARP study: a quantitative and qualitative evaluation of the short-term outcomes of housing and neighbourhood renewal**. *BMC Public Health* 2009, **9**:415.**Scottish Household Condition Survey Questionnaire** [http://surveynet.ac.uk/sqb/qb/surveys/shcs/96mainques.pdf]

2. Bajekal M, Primatesta P, Prior G: **Health Survey for England 2001**. London; 2003.

3. Attwood C, Singh G, Prime D, Creasey R: **2001 Home Office Citizenship Survey: people, families and communities**. In *Home Office Research Study. Volume 270*. London: Home Office; 2003

4. Stafford M, Nazroo J, Popay JM: **Tackling inequalities in health: evaluating the New Deal for Communities initiative**. *J Epidemiol Community Health* 2008, **62**:298–304.

Benzeval M, Der G, Ellaway A, Hunt K, Sweeting H, West P, Macintyre S: **Cohort Profile: West of Scotland 20-07 study: health in the community.** *Int J Epidemiol* 2009(38):1215-1223.

6. Macintyre S, Annandale E, Ecob R, Ford G, Hunt K, Jamieson B, MacIver S, West P, Wyke S: **The West of Scotland Twenty-07 Study: health in the community**. In *Readings for a new public health*. Edited by Martin C, McQueen D. Edinburgh: Edinburgh University Press; 1989:56-74.

7. Office for National Statistics SSD: **Living in Britain: results from the 2001 General Household Survey**. London; 2002.

8. Babb P: **Measurement of social capital in the UK**. In *Social and Welfare*. London; 2005.
